# Risk factors associated with progressive nerve fiber layer thinning in open-angle glaucoma with mean intraocular pressure below 15 mmHg

**DOI:** 10.1038/s41598-019-56387-x

**Published:** 2019-12-24

**Authors:** Jihei Sara Lee, Gong Je Seong, Chan Yun Kim, Sang Yeop Lee, Hyoung Won Bae

**Affiliations:** Department of Ophthalmology, Severance Hospital, Institute of Vision Research, Seoul, Korea

**Keywords:** Optic nerve diseases, Prognostic markers

## Abstract

The purpose of this study was to identify risk factors associated with progressive retinal nerve fiber layer(RNFL) thinning of open-angle glaucoma(OAG) in patients whose intraocular pressure(IOP) was maintained low with medical treatment. Based on a retrospective review of medical records, OAG patients with ≥60 months of follow-up and mean IOP below 15 mmHg were recruited. All eyes underwent IOP measurement with Goldmann applanation tonometer(GAT), standard automated perimetry(SAP), and cirrus optical coherence tomography(cirrus OCT) at 6 month or 1 year intervals. RNFL thinning was assessed using the Guided Progression Analysis(GPA) software. Forty-one eyes of 41 patients (mean age 54.9 ± 13.5) were followed up for 77.8 ± 7.8 months. GPA detected 20 eyes (48.8%) with progressive RNFL thinning(−1.5 ± 0.5 um/year), who were subsequently classified as the ‘rapid progression group.’ Those whose rate of change in RNFL thickness was slower than −1.00 µm/year was classified as the ‘slow progression group’ (n = 21, −0.0 ± 0.4 um/year, P < 0.001). Mean IOP after initiating therapy was 13.2 ± 1.1 mmHg in the rapid progression group and 13.1 ± 1.3 mmHg in the slow progression group (P = 0.300; 14.8 ± 10.0% vs. 19.6 ± 12.4% reduction, P = 0.155). Disc hemorrhage was found to more frequently occur in the rapid progression group (P = 0.001). Multivariate logistic regression analysis showed that patients with disc hemorrhage were at a higher risk for progressive RNFL thinning in OAG (OR 37.529 95% CI 2.915–483.140) after adjusting for baseline co-variates (P = 0.005). In conclusion, disc hemorrhage is associated with progressive RNFL thinning in OAG with well-maintained IOP. Factors other than IOP appear to also play a role in OAG progression.

## Introduction

Open-angle glaucoma(OAG) is defined as progressive optic nerve damage with open anterior chamber angle; it is characterized by the loss of retinal ganglion cells and associated morphologic changes to the optic nerve and retinal nerve fiber layer(RNFL)^1^. Today, spectral-domain (SD) optical coherence tomography(OCT) technology detects RNFL thickness with high sensitivity and reproducibility^2,3^. The longitudinal examination of RNFL thickness has allowed clinicians to identify progressive RNFL thinning before visual field defects appear.

Several studies to date have examined risk factors associated with progression of OAG. These studies have identified intraocular pressure(IOP) as the most important risk factor among others^4,5^. Further studies have also suggested that reduction of IOP suppresses the progression of the disease. Current clinical practice is therefore focused on lowering IOP in glaucomatous patients. However, it is not unusual in clinical setting to encounter patients who continue to show progression despite adequately controlled IOP.

While few studies have attempted to find factors involved in progression of treated OAG, most of them have considered those associated with functional progression^6,7^. In light of the above, the purpose of the present study was to identify risk factors of progressive RNFL thinning in OAG patients whose IOP was adequately reduced and maintained below 15 mmHg with medical therapy.

## Results

### Baseline characteristics

**Figure 1:**
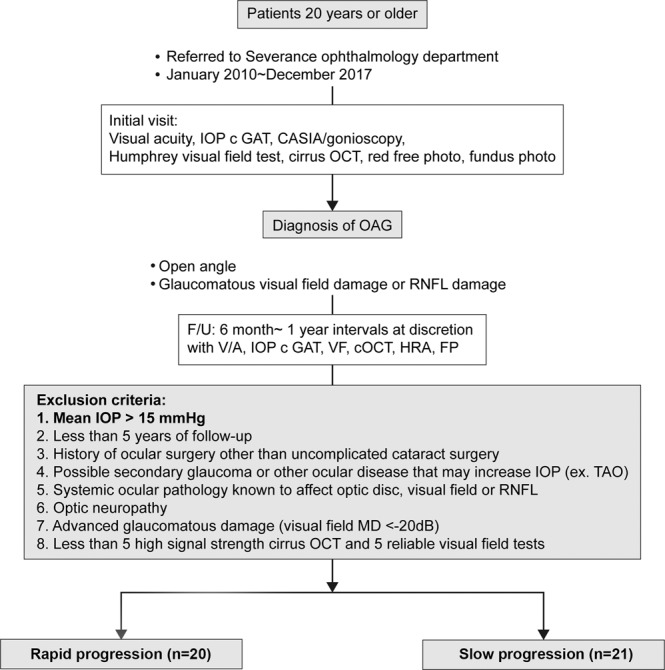
Flow chart of inclusion and exclusion of study participants. Patients older than 20 years, who were diagnosed with OAG, were followed every 6 months to 1 year. After excluding those whose average IOP were higher than 15 mmHg, they were categorized into rapid progression and slow progression groups based on the results of GPA analysis.

Forty-one eyes of 41 patients, who were diagnosed with open-angle glaucoma, were enrolled (Fig. 1). Table 1 demonstrates the baseline characteristics of the study population. The rapid progression group consisted of 20 eyes with mean age of 56.4 ± 11.8 years at the time of diagnosis (vs. 53.6 ± 15.1 years in the slow progression group, P = 0.518). Males made up 40.0% of the rapid progression group, whereas 66.7% of the slow progression group were males (P = 0.081). Seven patients in the rapid progression group (35%) and six patients in the slow progression group (28.6%) had pseudophakic eyes (P = 0.658). Two of the rapid progression patients (10%) and 1 of the slow progression patients (4.8%) have undergone cataract extraction operations during the follow-up period (P = 0.520). At baseline, the rapid progression and slow progression groups showed no statistically significant difference in mean deviation of the visual field test (−3.6 ± 3.3 dB for the rapid progression group vs. −4.7 ± 4.5 dB for the slow progression group; P = 0.406) and RNFL thickness (81.4 ± 7.7 µm for the rapid progression group vs. 76.7 ± 9.8 µm for the slow progression group; P = 0.095). RNFL thickness decreased at a rate of −1.5 ± 0.5 µm/year in the rapid progression group, whereas that in slow progression group showed 0.0 ± 0.4 µm/year of change on average. Follow-up period of the rapid progression group was 76.4 ± 8.0 months. No statistical difference in follow-up duration from the slow progression group was noted (79.3 ± 7.7 months, P = 0.245). The two groups did not show a statistically significant difference in axial length (24.4 ± 1.5 mm vs. 25.0 ± 2.0 mm, P = 0.313) or in refractive error (−1.8 ± 3.3 vs. −3.1 ± 4.6, P = 0.307). The maximum number of topical glaucoma medications as well as the types of medications did not differ between the two groups. Systemic conditions such as hypertension and diabetes mellitus did not differ between the two groups, either.

**Table 1:** Baseline clinical characteristics of open-angle glaucoma patients with and without RNFL thinning during follow-up period.

	Rapid progression (n = 20)	Slow progression (n = 21)	P
Age at diagnosis (year)	56.4 ± 11.8	53.6 ± 15.1	0.518^†^
Male (%)	8 (40.0)	14 (66.7)	0.081^‡^
Pseudophakia (%)	6 (30.0)	6 (28.6)	0.457^‡^
Axial length (mm)	24.4 ± 1.5	25.0 ± 2.0	0.313^†^
Refraction error (D)	−1.8 ± 3.3	−3.1 ± 4.6	0.307^†^
CCT (μm)	534.7 ± 30.9	550.0 ± 36.5	0.190^†^
Baseline MD (dB)	−3.6 ± 3.3	−4.7 ± 4.5	0.406^†^
Baseline PSD (dB)	3.5 ± 2.9	5.5 ± 4.6	0.093^†^
Average MD rate of change (dB/year)	−0.3 ± −0.4	0.1 ± 0.3	**0.004**^**†**^
Baseline RNFLT (μm)	81.4 ± 7.7	76.7 ± 9.8	0.095^†^
Average RNFL rate of change(μm/year)	−1.5 ± 0.5	0.0 ± 0.4	**<0.001**^**†**^
Follow-up (months)	76.4 ± 8.0	79.3 ± 7.7	0.245^†^
Disc hemorrhage (%)	10 (50.0)	1 (4.8)	**0.001**^**‡**^
**Topical medications**			
Maximum number of medications	1.4 ± 0.5	1.3 ± 0.5	0.668^‡^
β-blocker (%)	11 (55.0)	8 (38.1)	0.278^‡^
Brimonidine (%)	5 (25.0)	5 (23.8)	0.929^‡^
Prostaglandin analogue (%)	11 (55.0)	14 (66.7)	0.444^‡^
**Systemic medical conditions**			
Hypertension (%)	6 (30.0)	11 (52.4)	0.128^‡^
DM (%)	2 (10.0)	3 (14.3)	0.524^‡^
Systemic β-blocker (%)	1.0 (5.0)	3 (14.3)	0.317^‡^

Abbreviations: CCT, central corneal thickness; IOP, intraocular pressure; MD, mean deviation; RNFLT, retinal nerve fiber layer thickness; DM, diabetes mellitus.

P < 0.05 was considered statistically significant.

^†^Student t-test, ^‡^Chi-square test.

### Comparison of IOP changes

As illustrated in Table 2, the rapid progression group did not show a meaningful difference in the baseline intraocular pressure (15.8 ± 2.2 mmHg vs. 16.5 ± 2.5 mmHg, P = 0.300). After the initiation of medical therapy, both groups showed similar mean IOP; average IOP was 13.2 ± 1.1 mmHg in the rapid progression group and 13.1 ± 1.3 mmHg in the slow progression group (P = 0.638). The rapid progression group showed a decrease of 2.5 ± 1.9 mmHg (14.8 ± 10.0% reduction from baseline) from the baseline, whereas the slow progression group showed a reduction of 3.2 ± 2.6 mmHg (19.6 ± 12.4%, P = 0.180). No statistically significant difference was noted in the IOP reduction between the two groups (P = 0.155). IOP appeared to fluctuate to a similar degree in both groups (1.7 ± 0.7 mmHg for rapid progression group vs. 1.7 ± 0.8 mmHg for the slow progression group; P = 0.892). The peak IOP was also similar (16.6 ± 2.6 mmHg for the rapid progression group vs. 16.5 ± 2.4 mmHg for the slow progression group; P = 0.923). The number of IOP measurements and the measurement intervals did not differ between the two groups (18.3 ± 4.5 times vs. 17.0 ± 4.9 times, P = 0.386; 4.4 ± 1.1 months vs. 5.0 ± 1.3 months, P = 0.125).

**Table 2:** Comparison of IOP measurements during follow-up period in open-angle glaucoma patients with and without progressive RNFL thinning.

	Rapid progression (n = 20)		Slow progression (n = 21)		P
	Average ± SD	Range	Average ± SD	Range	
Baseline IOP	15.8 ± 2.2	11.0–20.0	16.5 ± 2.5	12.0–21.0	0.300
Mean IOP	13.2 ± 1.1	10.6–14.8	13.1 ± 1.3	10.3–14.9	0.638
IOP reduction	2.5 ± 1.9	−0.2–6.9	3.5 ± 2.2	−2.2–7.8	0.155
IOP reduction (%)	14.8 ± 10.0	−1.6–34.5	19.6 ± 12.4	−18.6–37.1	0.180
Peak IOP	16.6 ± 2.6	13.0–20.0	16.5 ± 2.4	13.0–21.0	0.923
IOP fluctuation	1.7 ± 0.7	0.6–3.4	1.7 ± 0.8	0.3–3.8	0.892
IOP measurements	18.3 ± 4.5	13–30	17.0 ± 4.9	9–31	0.386
Measurement interval (months)	4.4 ± 1.1	2.7–7.0	5.0 ± 1.3	2.7–8.9	0.125

Abbreviations: SD, standard deviation; IOP, intraocular pressure.

P < 0.05 was considered statistically significant.

### Logistic regression analysis of factors associated with structural progression

A statistical comparison of the two groups illustrated that disc hemorrhage was more frequent among the rapid progression group than the slow progression group (50.0% vs. 4.8%, P = 0.001). A univariate logistic regression analysis also identified disc hemorrhage alone as a potential factor associated with RNFL thinning in OAG with IOP below 15 mmHg (OR 20.000, 95% CI 2.235–178.938, P = 0.007). As demonstrated in Table 3, a multivariate logistic regression analysis showed that disc hemorrhage was significantly associated with progressive RNFL thinning in OAG patients with low IOP even after adjusting for the co-variates. According to the analysis, patients who showed disc hemorrhage during the course of satisfactory medical therapy that maintained low IOP had approximately 37 times greater risk of structural progression (OR 37.529 95% CI 2.915–483.140, P = 0.005) than those that did not show disc hemorrhage.

**Table 3:** Univariate and multivariate logistic regression analysis for risk factors associated with progressive RNFL thinning.

	Univariate		Multivariate	
	OR(95% CI)	P	OR(95% CI)	P
Age at diagnosis	0.984 (0.940–1.031)	0.508	1.012 (0.947–1.082)	0.719
Male	0.333 (0.093–1.192)	0.091	0.271 (0.049–1.497)	0.134
Pseudophakia	0.743 (0.199–2.779)	0.659		
Axial Length	1.222 (0.829–1.802)	0.310		
Refraction error	0.910 (0.759–1.090)	0.306		
CCT	1.014 (0.993–1.036)	0.190		
**IOP**				
Baseline IOP	1.157 (0.881–1.518)	0.294		
Mean IOP	0.878 (0.517–1.489)	0.629		
IOP reduction	1.254 (0.916–1.717)	0.157		
% IOP reduction	1.041 (0.981–1.104)	0.183		
IOP fluctuation	1.417(0.320–6.266)	0.646	1.217 (0.130–11.387)	0.864
Peak IOP	0.987 (0.767–1.270)	0.920		
Baseline RNFLT	0.940 (0.873–1.012)	0.099	1.060 (0.959–1.172)	0.256
**Visual field**				
Baseline PSD	1.163 (0.969–1.395)	0.104		
Baseline MD	0.932 (0.791–1.098)	0.399		
Disc hemorrhage	20.000 (2.235–178.938)	**0.007**	**37.529 (2.915–483.140)**	**0.005**
**Systemic medical conditions**				
Hypertension	0.390 (0.108–1.407)	0.150		
Diabetes mellitus	1.500 (0.223–10.077)	0.677		
Follow-up duration	0.952 (0.877–1.034)	0.242	0.921 (0.826–1.026)	0.921

Abbreviations: CCT, central corneal thickness; IOP, intraocular pressure; RNFLT, retinal nerve fiber layer thickness; PSD, pattern standard deviation; MD, mean deviation; DM, diabetes mellitus.

## Discussion

The present study investigated risk factors associated with progressive RFNL thinning in OAG patients when their IOP remained below 15 mmHg following medical therapy. The results have shown that disc hemorrhage was significantly associated with structural progression in OAG with low IOP.

Mechanisms of disc hemorrhage are still not clearly understood. Some researchers have postulated that disc hemorrhage results from the rupture of blood vessels at the level of the lamina cribrosa^8^. Nitta *et al*. suggested in their study that widening of RNFL defect subsequently causes the rupture^9,10^. Others have explained disc hemorrhage as a consequence of vessel damage from hemodynamic factors like systemic vascular disease and dysfunctional auto-regulation of the blood flow to the optic nerve head^11^. The damage, in turn, causes ischemic changes around the optic disc. In fact, migraine, low systolic blood pressure, use of oral β-blockers have been listed as risk factors for disc hemorrhage^12^. While the pathophysiology of disc hemorrhage remains uncertain, multiple studies to date have consistently identified disc hemorrhage as one of the most important risk factors in glaucoma development as well as progression^13,14^. The Collaborative Normal-Tension Glaucoma Study(CNTGS) group has also reported that disc hemorrhage is associated with development and progression of normal-tension glaucoma(NTG)^15^. Our study investigated into risk factors associated with progression of OAG when IOP is maintained low with medical therapy; those eyes that demonstrated structural progression despite successful medical therapy tended to have events of disc hemorrhage during the course of the disease.

In our analysis of OAG patients whose IOP was maintained below 15 mmHg, disc hemorrhage was found to be associated with progressive RNFL thinning. A multivariate analysis showed that patients who showed disc hemorrhage had approximately 37 times higher risk of structural progression in form of RNFL thinning despite low IOP. A similar outcome was reported in 2015 by Lee *et al*.^16^. They reported that VF progression in low-teen NTG patients was associated with disc hemorrhage, but none of the IOP-related factors. On the other hand, VF progression in high-teen patients was related to mean IOP. Our study strengthens reports of previous studies that non-IOP related factors like vascular insufficiency might be more central to the mechanism behind disease progression when IOP is low in glaucomatous eyes. Our study also cautiously suggests that there exists a risk factor beyond IOP that influences progression of NTG.

A number of studies have attempted to determine the sufficient degree of IOP reduction in glaucoma. Initially, the CNTGS suggested that lowering IOP by at least 30% from baseline was necessary to effectively slow down disease progression^4,5^. Recently, however, studies have suggested somewhat lower target IOP reduction. Aoyama *et al*. have recommended that the IOP be reduced by at least 20% from baseline or below 10 mmHg^17^. Other studies have also argued in the past that in order for progression rate to decrease, IOP reduction should be made at least 25% from the baseline^18^. In our study, the patient population achieved approximately 17.2 ± 11.4% reduction of IOP from the baseline. Admittedly, IOP reduction fell short of the target 20% for the patient population partly because the baseline IOP tended to be low (16.1 ± 2.4 mmHg), owing to the high prevalence of NTG among Asian ethnicity^19–21^. Although the difference was statistically insignificant, the rapid progression group achieved only 14.8 ± 10.0% reduction while the slow progression group showed 19.6 ± 12.4% reduction. In hindsight, even if the average IOP of the rapid progression group was maintained at around 13.2 ± 1.1 mmHg, additional IOP reduction might have been necessary in patients who showed progressive RNFL thinning during the follow-up period. Further reduction of IOP may have slowed progression. In other words, it is difficult to completely rule out the influence of higher IOP of the rapid progression group on faster RNFL thinning.

In fact, some studies have shown that aggressive reduction of IOP after the appearance of disc hemorrhage tended to slow the rate of visual field change in comparison to eyes with disc hemorrhage whose IOP was not significantly reduced^22^. Akagi *et al*. have also concluded in their study that RNFL thinning not only proceeds faster in quadrants with disc hemorrhage than in areas without disc hemorrhage, the rate of progression became even faster once disc hemorrhage appeared^23^. When treatment intensified to further reduce IOP in glaucomatous eyes that displayed disc hemorrhage, the rate of RNFL thinning significantly slowed down. However, it is possible medical therapy alone was inadequate to further decrease the IOP for our study population. Nevertheless, the current study investigated into risk factors associated with structural progression when IOP was maintained below 15 mmHg. As both progression and non-progression groups showed no statistically significant differences in IOP-related parameters, the results of our comparisons suggest that in cases where IOP is significantly reduced, IOP might not be a risk factor associated with disease progression.

### Study limitations

First, the study is inherently limited by its retrospective design. As the patient data were retrospectively collected, the chronological order between RNFL thinning and disc hemorrhage was not certain. Second, the results of the study may not be applicable to the general OAG patient population. The sample population comprised of those patients referred to a tertiary glaucoma clinic for more specialized management. The study population also comprised of only Korean population, among whom NTG is known to be more prevalent than OAG with high IOP. Further, the relatively small number of patients included in this study may have affected the results. However, when we retrospectively calculated the post-hoc power based on the assumption of a type I error equal to 0.05, the main outcome, disc hemorrhage, was found to have a sufficient power (97.2% in logistic regression analysis; data not shown). The required sample size for reliable detection was 21. The study results do need further confirmation with a larger number of patients. Third, using the average IOP as the main selection criterion may have led to inclusion of those patients who had large diurnal IOP variations as well as those who had fluctuations between follow-up periods our study. Fourth, the effect of cataract extraction operation on measurements of RNFL thickness was not accounted for. However, because the number of patients who have received uncomplicated cataract surgery is similar between the two groups, we believe the effect may have cancelled out and minimally affected the results of analyses. Fifth, it is possible that disc hemorrhage may have gone undetected in both the progression and non-progression group as most patients were followed up at clinic at 6 month intervals, and disc hemorrhage is reported to last for only 2 to 6 months^24^. However, we believe that while failure to detect disc hemorrhage between the follow-ups might have affected the frequency rates, exclusion of the events for both the rapid progression and slow progression groups would result in no bias. Lastly, the study did not take into account factors such as diastolic blood pressure of mean ocular perfusion pressure, which were previously identified as risk factors of progression in glaucoma^6^. Despite these limitations, a clear relationship between disc hemorrhage and progressive RNFL thinning in OAG with well-controlled IOP was observed in this study.

In conclusion, our study attempted to identify progression risk factors in OAG patients whose IOP was assumed to be well controlled in a clinical setting. The results showed a significant association between disc hemorrhage and RNFL thinning. In patients that showed disc hemorrhage, non-IOP factors such as vascular insufficiency may be more crucial to pathophysiology. Further studies are necessary to identify means to moderate these risk factors and help elucidate the pathophysiology of OAG.

## Methods

### Study population

Our study was approved by the Institutional Review Board of the Severance Hospital at Yonsei University, and adhered to the tenets of the Declaration of Helsinki. All participants gave written informed consent after explanation on the purpose and the procedure of the study. The patient population was chosen based on a retrospective, pre-recruitment chart review of patients who visited Glaucoma clinic at Severance hospital and were diagnosed with OAG from January of 2010 through December, 2017. Patients who were followed for at least five years and maintained average IOP below 15 mmHg were considered eligible for inclusion in the study. Those meeting any one of the following criteria were then excluded: (1) any history of ocular surgery other than uncomplicated cataract surgery, (2) possibility of secondary glaucoma or other ocular disease that may cause increase in IOP such as thyroid eye disease, (3) systemic ocular pathology known to affect optic disc, visual field or RNFL thickness, (4) advanced glaucomatous damage (i.e. visual field test with MD less than −20 dB by field analyzer measurement), and (5) less than 6 high signal strength (<6/10) cirrus OCT tests and 5 reliable visual field examinations (A “reliable” visual field test was defined to have ≤33% fixation losses and false negatives and ≤15% false-positives). In cases in which both eyes of a single subject were eligible, one eye was randomly chosen.

### Ophthalmologic evaluation

All patients underwent a complete ophthalmologic examination during an initial visit. The initial examination consisted of underlying medical history review, visual acuity, refraction, slit-lamp examination, gonioscopy, central corneal thickness(CCT) measurement, dilated fundus examination with a 90D lens, color disc stereophotography, red-free fundus photography, standard automated perimetry (24–2 SITA standard, Humphrey Field Analyzer II; Carl Zeiss Meditec, Inc., Dublin, California) and cirrus OCT(Carl Zeiss Meditec, Inc., Dublin, California). IOP was measured using Goldmann applanation tonometer (GAT; Haag-Streit model BQ-900; Haag-Streit, Inc., Bern, Switzerland). A diagnosis of OAG was made when a patient showed glaucomatous optic disc damage and corresponding visual field defects without any identifiable cause for optic nerve damage while gonioscopic examination revealed an open angle. After the initial visit, the patients were followed at 6 months to 1year intervals at discretion; during each visit, visual acuity, IOP, slit-lamp biomicroscopy, stereoscopic optic disc photography, visual field exam, red-free fundus photography and cirrus OCT were collected from the patients.

### Follow-up examinations of IOP and disc hemorrhage

The baseline IOP was taken as the average value of 3 repeated measurements taken during separate follow-up visits. The percentage reduction in IOP was defined as the difference between baseline IOP and mean IOP during follow-up divided by the baseline IOP. The standard deviation of all IOP collected during each individual’s follow-up period was taken to be IOP fluctuation. Optic disc hemorrhage was defined as hemorrhage located within one disc diameter of the optic disc border and not associated with optic disc edema, papillitis, diabetic retinopathy or retinal vein occlusion. Its presence was detected by masked assessment of optic disc stereophotographs. Two experienced graders evaluated stereophotographs while remaining masked to the subject’s identity and each other’s results. All photographs subject to examination were judged to be at least of adequate quality. Discrepancies were resolved by consensus or by consulting a third experienced grader.

### Determination of RNFL thinning

RNFL thickness was measured using the optic disc cube protocol of cirrus OCT version 6.0 software (Cirrus OCT, Carl Zeiss Meditec, Inc., Dublin, CA). The optic disc cube protocol takes 6 × 6 mm^2^ parapapillary region centered on the optic nerve head and collects 200 × 200 pixels to generate an RNFL map. Then, an algorithm called Guided Progression Analysis(GPA) was employed to detect progressive RNFL thinning. The algorithm aligned individual superpixels (4 × 4 pixels) and registers thickness at each of these superpixels. It set initial 2 OCT examinations as the baseline; then, it compared changes at individual superpixels from the baseline to the follow-up scans. In order to determine the rate of change in RNFL thickness, the aligned image series was subjected to a trend-based progression analysis. A linear regression analysis was performed between the RNFL thickness and follow-up time at individual superpixels of the RNFL thickness maps. A RNFL thickness change was defined significant when a significant trend was noted in 20 contiguous superpixels in the linear regression. Previously reported mean rates of global circumpapillary RNFL thickness change have ranged from −0.71um/year to −0.98um/year^25–27^. In our study, those patients, whose mean rates of global RNFL thickness change over the follow-up period were lower than −1.00 µm/year, were classified as the “rapid progression group.”(n = 20) Those whose rate of change remained higher than −1.00 µm/year during the follow-up period were categorized into the “slow progression group.”(n = 21) The slow progression group also included patients who showed age-related physiologic decline in RNFL at a rate between −0.1 and −0.3 µm/year^28^.

### Statistical analysis

All continuous data are presented as mean ± standard deviation. All categorical data were presented as actual number and percentage of the group. Comparisons of demographic and clinical characteristics between the progression and non-progression groups were made. Normally distributed continuous data were compared using the independent Student’s t-test while categorical data comparisons were performed using the chi-square test. Univariate and multivariate logistic regression models were used to identify the risk factors associated with structural progression in OAG. Those parameters that showed P values less than 0.1 during the univariate step were carried forward to the multivariate step. Adjusted hazard ratios (HRs) with 95% confidence intervals (CI) were calculated for both steps. All statistical analyses were performed using SPSS version 16.0 (SPSS Inc., Chicago, IL, USA). A p-value less than 0.05 was considered statistically significant.

## Data Availability

The datasets generated during and/or analyzed during the current study are available from the corresponding author on reasonable requests. No restrictions on the availability of materials or information.
